# Insights into melatonin-induced photosynthetic electron transport under low-temperature stress in cucumber

**DOI:** 10.3389/fpls.2022.1029854

**Published:** 2022-11-03

**Authors:** Pei Wu, Yadong Ma, Golam Jalal Ahammed, Baoyu Hao, Jingyi Chen, Wenliang Wan, Yanhui Zhao, Huimei Cui, Wei Xu, Jinxia Cui, Huiying Liu

**Affiliations:** ^1^ Department of Horticulture, Agricultural College, Shihezi University, Shihezi, China; ^2^ The Key Laboratory of Special Fruits and Vegetables Cultivation Physiology and Germplasm Resources Utilization in Xinjiang Production and Construction Group, Shihezi University, Shihezi, China; ^3^ College of Horticulture and Plant Protection, Henan University of Science and Technology, Luoyang, China

**Keywords:** cucumber, low temperature, melatonin, OJIP, MR_820_ signal, JIP-test

## Abstract

In this study, the differences in chlorophyll fluorescence transient (OJIP) and modulated 820 nm reflection (MR_820_) of cucumber leaves were probed to demonstrate an insight into the precise influence of melatonin (MT) on cucumber photosystems under low temperature stress. We pre-treated cucumber seedlings with different levels of MT (0, 25, 50, 100, 200, and 400 μmol · L^-1^) before imposing low temperature stress (10 °C/6 °C). The results indicated that moderate concentrations of MT had a positive effect on the growth of low temperature-stressed cucumber seedlings. Under low temperature stress conditions, 100 μmol · L^-1^ (MT 100) improved the performance of the active photosystem II (PSII) reaction centers (PIabs), the oxygen evolving complex activity (OEC centers) and electron transport between PSII and PSI, mainly by decreasing the L-band, K-band, and G-band, but showed differences with different duration of low temperature stress. In addition, these indicators related to quantum yield and energy flux of PSII regulated by MT indicated that MT (MT 100) effectively protected the electron transport and energy distribution in the photosystem. According to the results of *W_O-I_* ≥ 1 and MR_820_ signals, MT also affected PSI activity. MT 100 decreased the minimal value of MR/MR_O_ and the oxidation rate of plastocyanin (PC) and PSI reaction center (P700) (*V_ox_*), while increased △MR_slow_/MR_O_ and deoxidation rates of PC^+^ and P_700_^+^ (*V_red_*). The loss of the slow phase of MT 200 and MT 400-treated plants in the MR_820_ kinetics was due to the complete prevention of electron movement from PSII to re-reduce the PC^+^ and P700 ^+^. These results suggest that appropriate MT concentration (100 μmol · L^-1^) can improve the photosynthetic performance of PS II and electron transport from primary quinone electron acceptor (Q_A_) to secondary quinone electron acceptor (Q_B_), promote the balance of energy distribution, strengthen the connectivity of PSI and PSII, improve the electron flow of PSII *via* Q_A_ to PC^+^ and P_700_^+^ from reaching PSI by regulating multiple sites of electron transport chain in photosynthesis, and increase the pool size and reduction rates of PSI in low temperature-stressed cucumber plants, All these modifications by MT 100 treatment promoted the photosynthetic electron transfer smoothly, and further restored the cucumber plant growth under low temperature stress. Therefore, we conclude that spraying MT at an appropriate concentration is beneficial for protecting the photosynthetic electron transport chain, while spraying high concentrations of MT has a negative effect on regulating the low temperature tolerance in cucumber.

## Introduction

Cucumber (*Cucumis sativus* L.), an important economic and nutritional crop, is cultivated in diverse climatic regions around the world, although it originated from tropical and subtropical areas. Due to high sensitivity to environmental factors, cucumber is often subjected to multiple environmental stresses, especially low temperature (0 °C to 15 °C) when grown in cool seasons (Chinnusamy et al., 2010; Theocharis et al., 2012). The adverse effects of low temperature on cucumber plant growth and development are mainly manifested through severe damage to photosynthetic components and efficiency (Ensminger et al., 2006; Ploschuk et al., 2014; Wu et al., 2020; Zhang et al., 2020; Lee et al., 2021). The deleterious effects on photosynthesis caused by low temperature are multifaceted, on the one hand, low temperature directly decreases the chlorophyll content and disrupts the chloroplast structure, resulting in the reduction of light energy capture that can be absorbed and utilized by plants (Liu et al., 2018); besides, low temperature indirectly reduces the carbon dioxide (CO_2_) fixation capacity by reducing the sensitivity of stomata to CO_2_ (Xiong et al., 2015; Wu et al., 2020). Low temperature stress also exacerbates an imbalance between the energy absorption by photosystems and the metabolic sink of plants, and the imbalance activates the redox sensor within the photosynthetic electron transport chain, thereby regulating photophysical, photochemical and metabolic processes by photosynthetic electron transport in the chloroplast (Ensminger et al., 2006; Ruelland et al., 2009). Therefore, it is necessary to explore strategies to protect the photosystem damage and improve the photosynthesis of plants under low temperature stress. In recent years, studies on the application of exogenous plant growth regulators and/or signaling agents including nitric oxide (NO), brassinolide (BR), hydrogen sulfide (H_2_S), glutathione (GSH), calcium (Ca^2+^), and melatonin (MT) have provided a theoretical basis on protecting photosystems and improving the photosynthetic capacity of plants under abiotic stress (Cui et al., 2011; Zhou et al., 2018; Corpas, 2019; Wu et al., 2020; Zhang et al., 2020; Feng et al., 2021).

Since its discovery in plants, MT has attracted more and more attention from plant scientists due to its involvement in plant growth, development, photosynthesis, rooting, seed germination, biotic, and abiotic stress responses (Arnao and Hernández-Ruiz, 2014; Reiter et al., 2015; Debnath et al., 2019; Khan et al., 2020; Sun et al., 2020; Li et al., 2021; Wang et al., 2022). The efficacy of MT in reactive oxygen species (ROS) scavenging and antioxidant defense responses are the two major mechanisms to cope with major abiotic stresses (Sun et al., 2020; Tiwari et al., 2020). Notably, MT is involved in regulating the functions of photosynthetic apparatus and photochemical reactions. For instance, MT treatment increases the maximal quantum yield of PSII (Fv/Fm), the actual photochemical efficiency of PSII (Y(II)), electron transport rate (ETR) and photochemical quenching (qP), while it decreases nonphotochemical quenching (NPQ) to increase the high-temperature tolerance of tomato plants (Jahan et al., 2021). Furthermore, exogenous MT can protect maize from drought stress by inhibiting excessive ROS accumulation, while promoting glutathione (GSH) metabolism, calcium (Ca^2+^) signals transduction, and jasmonic acid (JA) biosynthesis (Zhao et al., 2021). Notably, exogenous MT has also been reported to improve the photochemical processes of PSII, by directly increasing antioxidant enzyme activities, leading to altered metabolism in bermudagrass under cold stress (Fan et al., 2015). However, detailed and comprehensive information on the MT-induced alleviation of low temperature-inhibited photosynthetic energy allocation and electron transport in cucumber is still unavailable.

The energy captured by chloroplast is mostly used for photochemical reactions (Wang et al., 2020). After excitation, the reaction center chlorophylls P680 in PSII and P700 in PSI are photo-oxidized, allowing electron transport from H_2_O to NADP^+^ along with electron transporters complexes (cytochrome *b_6_f* complex (cyt *b_6_f*) and quinone acceptors of PSII (Q_A_, Q_B_, plastocyanin (PC)), which are finally oxidized to produce the adenosine-triphosphate (ATP) and reduced coenzyme II (NADPH) (Shikanai, 2011; Krieger-Liszkay and Shimakawa, 2022). In addition, a part of the energy that cannot be utilized for the photochemical reaction is dissipated by heat (internal conversion) and fluorescence, in which the energy used for fluorescence accounts for 3-5% of the total energy absorbed by chlorophyll (Strasser et al., 1995). Fortunately, as a sensitive, non-destructive, rather quickly, and reliable tool, chlorophyll *a* fluorescence provides convenience for investigating the ecophysiological indexes of plant stress (Strasser et al., 2004; Wang et al., 2020; Chen et al., 2021). The prompting fluorescence transient (OJIP) and modulated 820 nm reflection (MR_820_) signal are simultaneously measured by a new instrument (M-PEA) which are informative in evaluating the photochemical efficiency and the characteristics of the components related to photosynthetic electron transport (Strasser et al., 2010; Stirbet and Govindjee, 2011; Chen et al., 2016; Guo et al., 2020). OJIP transient analyses have revealed that abiotic stress including salt, cold, and high temperature could change the thylakoid component processes, light utilization efficiency, and excitation energy dissipation, and also reduce the stability of the photosynthetic system and the connectivity between PS1 and PSII in plants (Hu et al., 2018; Snider et al., 2018; Chen et al., 2021). The procedure for biophysical interpretation of fluorescence transient provides convenience for our research.

In this study, we hypothesized that MT could affect photosynthetic electron transport in low temperature-stressed cucumber plants to confer low temperature tolerance. Particularly, we aimed to get a better insight into the precise influence of MT on cucumber photosystems. Accordingly, cucumber seedlings pre-treated with different concentrations of MT were subject to low temperature stress and used to simultaneously measure the OJIP and MR_820_ signals. Based on the “theory of energy fluxes in biomembranes”, we investigated the effect of MT on the photochemical efficiency and the characteristics of the components related to photosynthetic electron transport using the JIP-test method. The results obtained provide valuable insight into the mechanism of MT-induced photosynthetic regulation which can be a reference for further understanding the regulatory pathway of MT-induced enhanced low temperature tolerance in cucumber plants.

## Materials and methods

### Plant materials and chemical treatment

The cucumber (*C. sativus* L.) cultivar ‘Jinyan No. 4’ was used for the current experiment. The seedlings were transplanted in pots (12-cm-diameter, with one seedling per pot) filled with the specified substrate (peat: vermiculite, 2: 1, v/v) and raised in an incubator at a temperature of 25/18 °C (day/night), the light intensity of 300 μmol · m^-2^ · s^-1^ (PPFD), and relative humidity of 75%-80%, and photoperiod of 14 h/10 h (day/night). The chemical treatments were conducted when the third true leaves were expanded. Twenty-four seedlings were divided into 6 groups and pre-treated with distilled water (LT) or different concentrations of melatonin (MT, purchased from Yuanye Company, China) such as 25 μmol · L^-1^ (MT 25), 50 μmol · L^-1^ (MT 50), 100 μmol · L^-1^ (MT 100), 200 μmol · L^-1^ (MT 200), and 400 μmol · L^-1^ (MT 400) and cultured at 25 °C, 0 μmol · m^-2^ · s^-1^ (PPFD) and humidity of 75% for 4 h, and then 300 μmol · m^-2^ · s^-1^ was restored. Twenty-four hours after the distilled water or chemical treatments, low temperature treatment (temperature of 10/6 °C (14 h-day/10 h-night cycle), light intensity of 100 μmol · m^-2^ · s^-1^, and relative humidity of 70%-75%) was initiated. And the prompting chlorophyll *a* fluorescence transient (OJIP) and modulated 820-nm reflection (MR_820_) signal were measured in the mature leaves (the second leaves from the bottom) of cucumber plants under low temperature stress at 24 h and 48 h.

### Phenotype of cucumber seedlings

We captured the pseudo color pictures of the maximal quantum yield of PSII (Fv/Fm) and the actual phenotype photos of cucumbers after low temperature stress for 72 h. And the Imaging-PAM-2500 (IMAG-MAX; Walz, Germany) was used to detect the value of Fv/Fm according to Zhang et al. (2020).

### Measurement of OJIP transient and MR_820_ signal

The cucumber plants were initially dark adapted for two hours by putting them in a dark incubator along with attachments of special plastic clips to the leaves. And then the OJIP and MR_820_ signal were simultaneously detected using M-PEA (Hansatech, Norfolk, UK) according to Zhou et al. (2019). The OJIP transients were induced by a saturating light pulse of 3000 μmol · m^-2^ · s^-1^ and recorded during a 5 s light pulse. Fluorescence values at 0.02 ms and 0.7 ms were considered to be the first reliable value of OJIP and MR_820_ signals, respectively. Then the JIP-test was used to analyze the OJIP and MR_820_ signals according to the method of Strasser et al. (2004). A series of data had been mentioned in the article including the performance of active reaction centers (RCs) (PIabs), potential activity of photosynthetic system (Fv/Fo), standardized variable fluorescence at J point (*V_J_*), the energy flux of per active RC (RE_O_/RC, TR_O_/RC, ABS/RC, ET_O_/RC, and DI_O_/RC), quantum yield (φ_Po_, φ_Eo_, φ_Ro_), flux ratio (ψ_Eo_, δ_Ro_), normalized total complementary area (Sm), and closing rate of PSII RCs (Mo). To further estimate the electron transport of the photosynthetic system, the O-P, O-K, O-J, and O-I periods were calculated by double normalization: *V_t_* = (*F_t_* – *F_O_*)/(*F_M_* – *F_O_*), *W_O-K_* = (*F_t_* – *F_O_*)/(*F_K_* – *F_O_*), *W_O-J_* = (*F_t_* – *F_O_*)/(*F_J_* – *F_O_*), and *W_O-I_* = (*F_t_* – *F_O_*)/(*F_I_* – *F_O_*). The fluorescence differences between MT treatments and LT were determined in the L-band, K-band, and G-band and calculated as: *ΔW_O-K_* =[*W_O-K_*_(treatment)_ – *W_O-K_*_(control)_], *ΔW_O-J_* =[*W_O-J_*_(treatment)_ – *W_O-J_*_(control)_], and *ΔW_O-I_* =[*W_O-I_*_(treatment)_ – *W_O-I_*_(control)_], respectively (Strasser et al., 2004; Silva Dalberto et al., 2017). M_O_ was calculated as: M_O_ = 4 (*F_270μs_* – *F_O_*)/(*F_M_* – *F_O_*); OEC centers was calculated as: OEC centers = [1 –(*V_K_*/*V_J_*)]_treatment_/[1 – (*V_K_*/*V_J_*)]_control_ (Guo et al., 2020).

Upon exclusion of the interference of other factors on the light reflection at 820 nm, the MR_820_ signals were represented by MR/MRo (Guo et al., 2020). MR_O_ represents the first reliable value of the MR/MRo (at 0.7 ms). Based on the MR/MR_O_ curve, we analyzed the redox state of PSI electron carriers of cucumber seedlings: plastocyanin (PC) and PSI reaction center (P_700_) were oxidized by the initial light (corresponding to the decreased fraction of MR/MR_O_, which can be represented by △MR_fast_/MR_O_) and followed reduction (corresponding to the increased fraction of MR/MR_O_, which can be represented by △MR_slow_/MR_O_) (Schansker et al., 2003; Strasser et al., 2010). The redox rates of PC and P_700_ are denoted by *V_ox_* and *V_red_*, respectively. According to Guo et al. (2020), the following formulae were used for various calculations: △MR_fast_/MR_O_ = (MR_O_ – MR_min_)/MR_O_, △MR_slow_/MR_O_=(MR_max_ – MR_min_)/MR_O_, *V_ox_* = △MR/△t = (MR_2 ms_ –MR_0.7 ms_)/(1.3 ms), and the calculation formula of *V_red_*.

### Statistical analysis

Statistical analyses were performed using variance analysis (ANOVA). The values were presented by the means ± SE of three replicates and the *P* < 0.05 was considered to be significantly different.

## Results

### MT-induced changes in phenotypic and fluorescence parameters in response to low temperature

**Figure 1:**
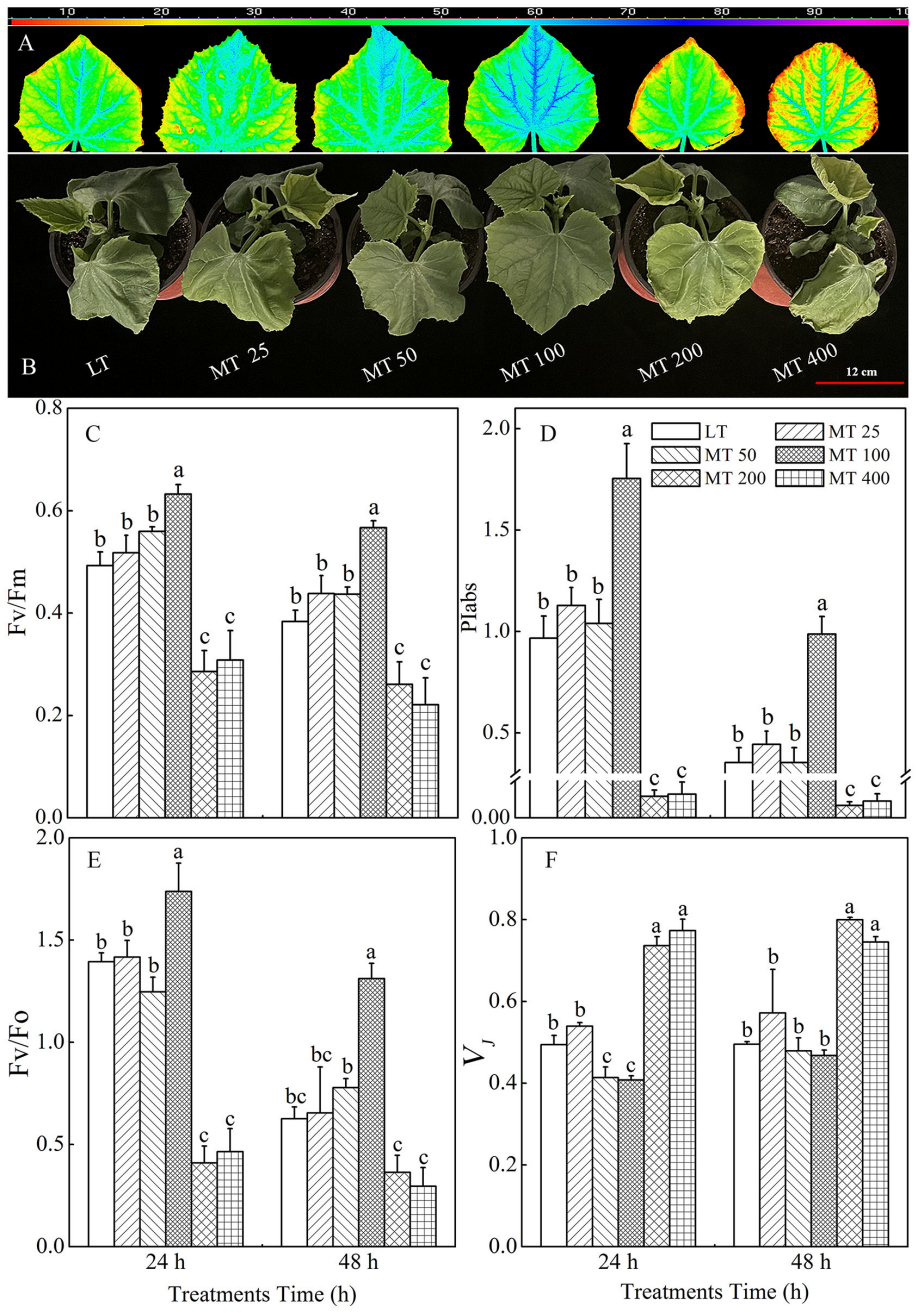
The image of Fv/Fm **(A)**, phenotype **(B)**, and the value of Fv/Fm **(C)**, PIabs **(D)**, Fv/Fo **(E)**, and *V_J_* **(F)** of cucumber seedlings as affected by different melatonin (MT) levels under low temperature stress. The values were represented by the means ± SE of three replicates. The same letters denoted that there are no significant differences at *P* < 0.05 according to Duncan’s test.

The phenotype of cucumber seedlings was significantly changed by different concentrations of MT under low temperature conditions (**Figure 1**). In comparison with the LT treatment, MT 50 and MT 100 treatments, especially the MT 100 treatment noticeably ameliorated the wilting phenotype and visible cold injuries, while MT 200 and MT 400 aggravated cold-induced damage to cucumber seedlings (**Figure 1**).

The changes in Fv/Fm, PIabs, Fv/Fo, and *V_J_* in cucumber plants treated with different MT concentrations under low temperature stress are shown in **Figures 1A, C–F**. The Fv/Fm was significantly increased with MT 100 treatment by 28.4% and 47.7% under low temperature stress for 24 h and 48 h, respectively, when compared with LT treatment (**Figure 1C**). The value of PIabs increased by 81.6% and 179.2% in ‘MT 100’-treated plants under low temperature stress for 24 h and 48 h, respectively when compared with LT. However, MT 200 and MT 400 treatments significantly decreased the PIabs (**Figure 1D**). In addition, Consistent with the Fv/Fm quantitative values (**Figure 1C**), the pseudo color image of Fv/Fm in **Figure 1A** showed the same trend. Fv/Fo represents the potential activity of the photosynthetic system, and *V_J_* reflects the closure degree of the active RCs of photosystem II (PSII). Under low temperature stress, MT significantly altered the value of Fv/Fo and *V_J_* in the cucumber leaves (**Figures 1E, F** **)**. The ‘MT 100’-treated plants had higher, while MT 200 and MT 400 plants had lower Fv/Fo in both 24 h and 48 h of low temperature stress than the LT-treated plants. In addition, MT 50 and MT 100 significantly decreased while the MT 200 and MT 400 treatments significantly increased the *V_J_* when compared with LT treatment.

### Effects of different levels of MT on the OJIP transient of cucumber plants under low temperature stress

#### Prompting fluorescence transient (OJIP) and the relative variable fluorescence (*V_t_*)

**Figure 2:**
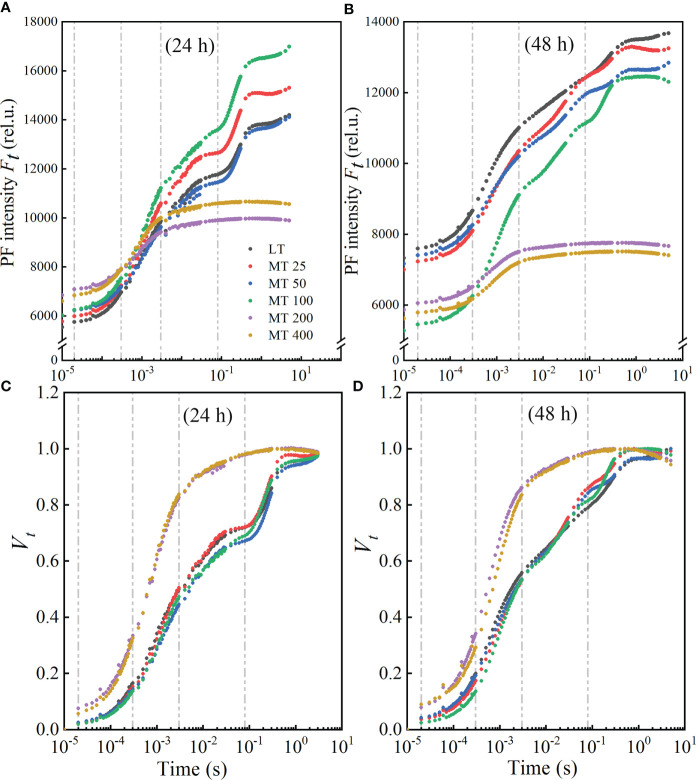
Effect of different melatonin (MT) concentrations on the induction of fluorescence transient (OJIP) of the cucumber seedlings under low temperature stress. The OJIP transients after low temperature stress for 24 h **(A)** and 48 h **(B)**; Normalized transients of OJIP in cucumber seedlings after low temperature for 24 h **(C)** and 48 h **(D)**. The *V_t_* was calculated as *V_t_* = [(*F_t_* − *F_O_*)/(*F_M_* − *F_O_*)].

OJIP transients of cucumber seedlings treated with different concentrations of MT under low temperature stress were presented in **Figure 2**. As shown in **Figures 2A, B**, the traditional J, I, and P points (2 ms, 30 ms, and approximately 300 ms, respectively) were delayed to J point for 3 ms, I point for 80 ms, and P point did not reach the real maximum value under low temperature stress in our study. Clearly, treatments with different MT concentrations exhibited different influences on the OJIP transients. The OJIP transients of cucumber seedlings that were treated with LT, MT 25, MT 50, and MT 100, showed a typical shape, while MT 200 and MT 400 treatments significantly changed OJIP shape under low temperature stress. The highest point of the OJIP curve (*F_p_*) decreased progressively with the extension of stress time (**Figures 2A, B** **)**. Compared with LT, MT treatments (MT 25, MT 50, MT 100, MT 200, and MT 400) significantly increased the *F_O_* under cold stress for 24 h, while a significant decrease in *F_O_* was observed after 48 h of stress. The MT 100-treated plants exhibited a higher *F_p_* level in 24 h and a more normal characteristic curve in stress for 48 h than LT treatment. In addition, MT 100 significantly increased the *Fp* under stress for 24 h, while significantly decreased the *Fo* under stress for 48 h when compared with LT treatment. The K-step was increased by the five MT treatments under 24 h of low temperature stress, while decreased by these MT treatments under 48 h of low temperature stress (**Figures 2A, B** **)**.

The double normalized OJIP curves from *F_O_* to *F_M_* were presented as *V_t_* (**Figures 2C, D** **)**, and to assess the characteristics of OJIP more clearly. Compared with the LT, the normalized OJIP curves of five MT concentrations-treated plants showed apparent and variable changes. The K-step and J-step decreased at MT 25, MT 50, and MT 100 treatments, while increased drastically at MT 200 and MT 400 treatments when compared with LT under low temperature conditions. In comparison with LT, different concentrations of MT (MT 25, MT 50, and MT 100) treatments led to a lower I-step under stress for 24 h, while a higher I-step under stress for 48 h. But the J-step and I-step were always the highest in MT 200 and MT 400 treatments under low temperature conditions (**Figures 2C, D** **)**.

#### The L-band of MT-pretreated cucumber plants under low temperature stress

**Figure 3:**
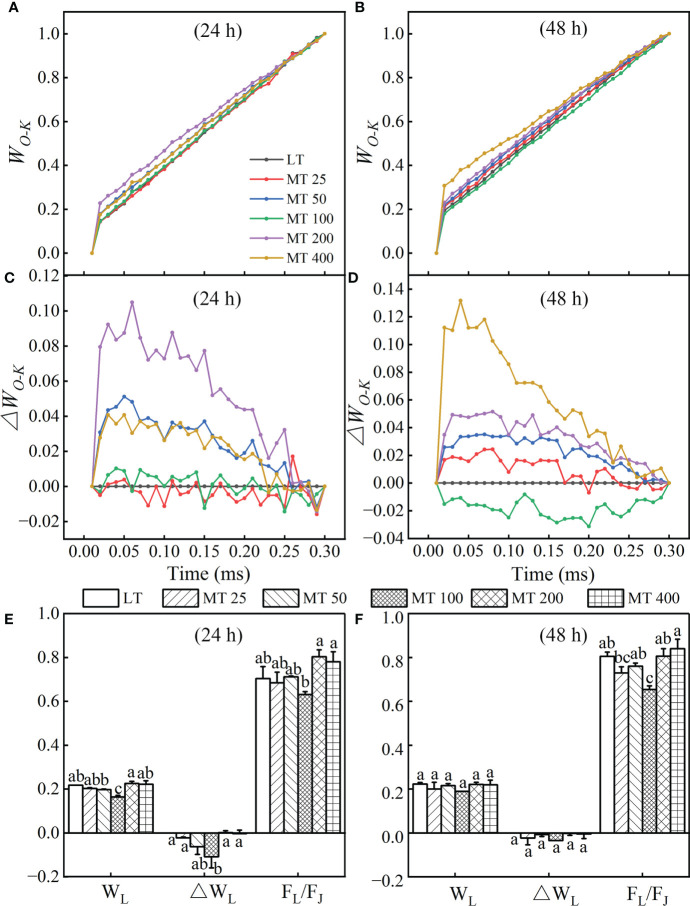
Effect of different melatonin (MT) concentrations on the L-band of low temperature-stressed cucumber plants. The OJIP kinetics normalized by O and K points, and calculated as: *W_O-K_* = (*F_t_* – *F_O_*)/(*F_K_* – *F_O_*).The difference kinetics *ΔW_O-K_* was calculated as *ΔW_O-K_* = *W_O-K_*_(treatment)_ – *W_O-K_*_(control)_. **(A, C)** and **(B, D)** represent low temperature stress for 24 h and 48 h, respectively. The W_L_, ΔW_L_ and F_L_/F_J_ values of MT-pretreated cucumber plants at the low temperature stress for 24 h **(E)** and 48 h **(F)**. The values were represented by the means ± SE. The same letters denoted that there are no significant differences at *P* < 0.05 according to Duncan’s test.

The L-band was analyzed to evaluate the aggregation between different components of PSII or the connectivity of energy transfer between antenna pigment and PSII active RC (Strasser et al., 2004) in cucumber leaves. The OJIP curves of each treatment were normalized by O- and K-point to show L-band, as *W_O-K_* kinetics (**Figures 3A, B** **)** and the difference kinetics *ΔW_O-K_* (**Figures 3C, D** **)** in the linear time variation from 0 to 300 μs. It showed that there were no differences in L-band between MT 25, MT 100 and LT treatments at 24 h (**Figure 3C**) of low temperature stress, while MT 100 decreased L-band obviously at 48 h (**Figure 3D**) of low temperature stress. However, MT 200 and MT 400 always increased the low temperature-stressed L-band of cucumber seedlings when compared with LT treatment (**Figures 3C, D** **)**. Under low temperature conditions, it is clear that MT 100 obviously changed the values of W_L_, ΔW_L_ and F_L_/F_J_ when compared with LT (**Figures 3E, F** **)**. Specifically, there was no significant difference between LT- and MT-treated cucumber seedlings in W_L_ and ΔW_L_, while MT 100 significantly decreased the F_L_/F_J_ at 48 h of low temperature stress. This suggests that MT-caused the change in L-band because of the increase of the J-step and the decrease in the L-step at stress for 24 h, while only the increase of the J-step at stress for 48 h.

#### The K-band of MT-pretreated cucumber plants under low temperature stress

**Figure 4:**
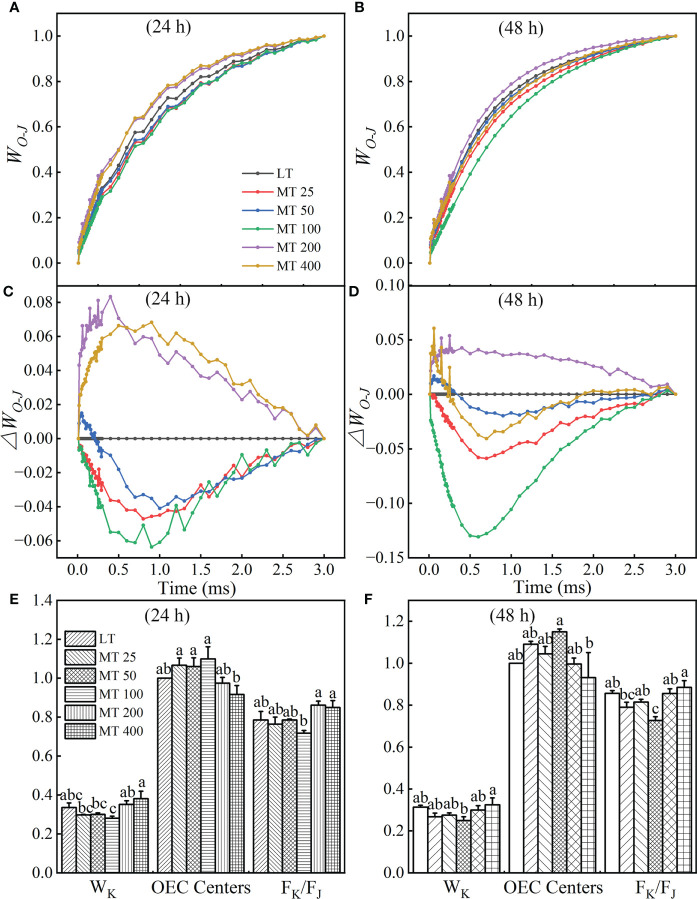
Effect of different concentrations of melatonin (MT) on the K-band of cucumber plants under low temperature stress. The OJIP curves were normalized by O and J points as *W_O-J_* = (*F_t_* – *F_O_*)/(*F_J_* – *F_O_*), and the difference kinetics *ΔW_O-J_* = *W_O-J_*_(treatment)_ – *W_O-J_*_(control)_. **(A, C)** and **(B, D)** represent low temperature stress for 24 h and 48 h, respectively. Effect of different levels of MT on the values of W_K_, OEC centers and F_k_/F_J_ at 24 h **(E)** and 48 h **(F)** of low temperature stress. The values were represented by the means ± SE. The same letters denoted that there is no significant difference at *P* < 0.05 according to Duncan’s test. Data are presented as the means of three biological replicates.

The OJIP curves were normalized by O and J points to show the K-band and were presented by *W_O-J_* (**Figures 4A, B** **)** and *ΔW_O-J_* (**Figures 4C, D** **)**. The *ΔW_O-J_* showed that the five MT treatments induced the occurrence of the K-band. Compared with LT, MT 25, MT 50, and MT 100 treatments significantly decreased, while MT 200 and MT 400 treatments increased the K-band under low temperature stress (**Figures 4C, D** **)**. In addition, compared with LT, only MT 100 treatment decreased the value of W_K_ and F_K_/F_J_ of cucumber plants under low temperature stress. The OEC center was increased by MT 100 treatment at a certain degree (**Figures 4E, F** **)**, which is highly consistent with the trend of *ΔW_O-J_* under low temperature stress. These results corroborated that MT 100 treatment can effectively protected the part of the active OEC centers.

#### The G-band of MT-pretreated cucumber plants under low temperature stress

**Figure 5:**
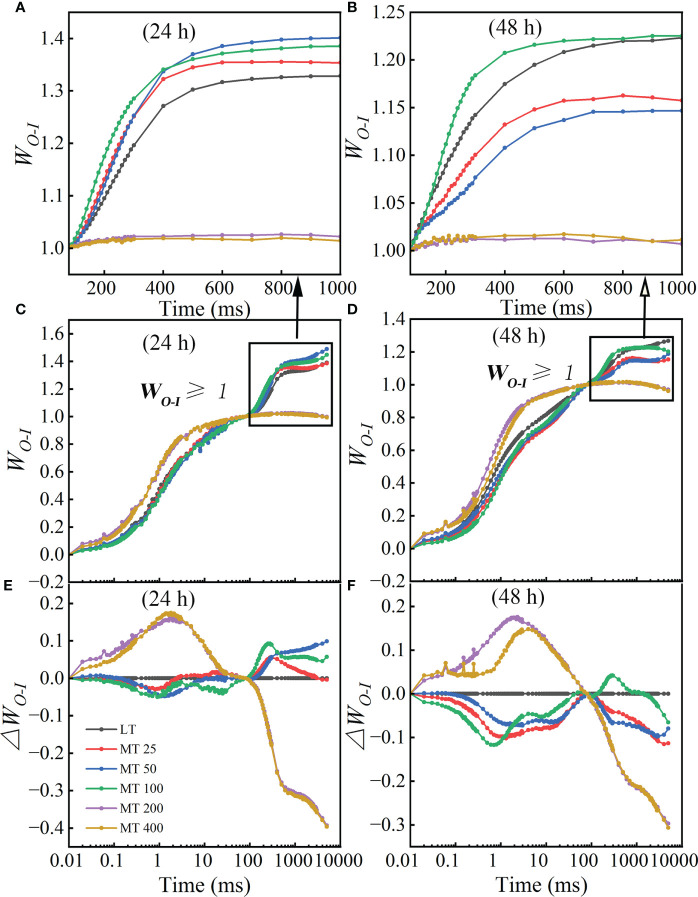
Different concentrations of melatonin (MT) induced the change in G-band shape in cucumber plants under low temperature stress. **(A, B)** The W_*O-I*_ curves from 80 ms to 1000 ms after 24 h and 48 h of low temperature-stressed cucumber seedlings. **(C, D)** The OJIP curves were normalized by O and I points as W_*O-I*_ = (F_*t*_ – F_*O*_)/(F_*I*_ – F_*O*_). **(E, F)** The difference kinetics calculated as D*_WO-I_* = W_*O-I (treatment)*_ – W_*O-I (control)*_ in a logarithmic time scale. Data are presented as the means of three biological replicates.

At the low temperature stress conditions, the normalizations and corresponding subtractions (difference kinetics) of OJIP curves from O to I point (80 ms) were presented in **Figures 5C–F**, as well as *W_O-I_* ≥ 1 plotted in the linear 80-1000 ms to show the IP phase (**Figures 5A, B** **)**. *ΔW_O-I_* represented the effects of different MT concentrations on the G-band. The results showed that the G-band of MT 25, MT 50, and MT 100 treatment was lower than LT, while MT 200 and MT 400 had higher G-band than LT treatment in low temperature-stressed cucumber plants (**Figures 5E, F** **)**. The maximum amplitude of the *W_O-I_* ≥ 1 curve is negatively correlated with the pool size of the terminal electron receptor on the PSI receptor side; specifically, the small amplitude corresponds to the strong inhibition effect on the pool size (Guo et al., 2020). Compared with LT, the amplitude of *W_O-I_* curves was significantly increased to various degrees by MT 25, MT 50, and MT 100 treatments, while significantly decreased by MT 200 and MT 400 treatments after low temperature stress for 24 h (**Figure 5A**). While only MT 100 treatment increased the amplitude, and the other treatments decreased the amplitude of *W_O-I_* ≥ 1 when compared with LT after low temperature stress for 48 h (**Figure 5B**).

### Effect of different MT concentrations on the JIP- test parameters of PSII

#### Specific fluxes per active RC

**Figure 6:**
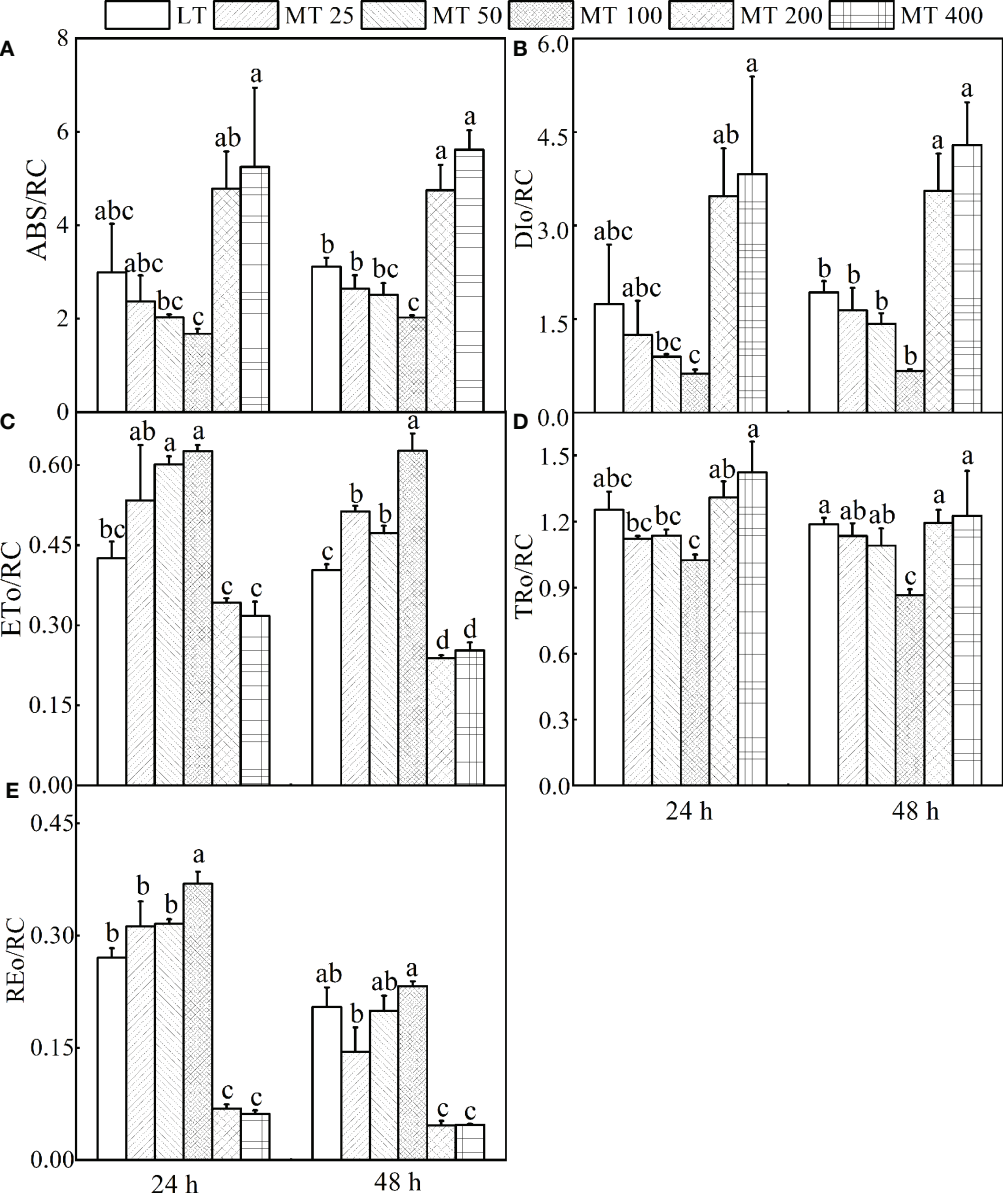
Parameters derived from OJIP transients of cucumber plants treated with different concentrations of melatonin (MT) under low temperature stress. **(A)** The energy absorbed by each active reaction center (RC). **(B)** The energy dissipated by each active RC. **(C)** The energy flux transferred by each active RC. **(D)** Excitation energy flux captured by each active RC. **(E)** The electron transport from QA- to the PSI electron acceptors by each RC. The values were represented by the means ± SE. The same letters denoted that there is no significant difference at P < 0.05 according to Duncan's test.

It is interesting to find out if MT influences the specific fluxes per active RC. The energy absorbed and dissipated by active RC (ABS/RC and DIo/RC), and excitation energy flux captured by each active RC (TRo/RC) were significantly decreased by MT 100, while increased by MT 200 and MT 400 treatments relative to LT treatment (**Figures 6A, B, D** **)**. In comparison with LT, an increase of energy flux transferred by each active RC (ETo/RC) and electron transport from Q_A_^-^ to the PSI electron acceptors by each RC (REo/RC) was observed in MT 100 treated plants (**Figures 6C, E** **)**.

**Figure 7:**
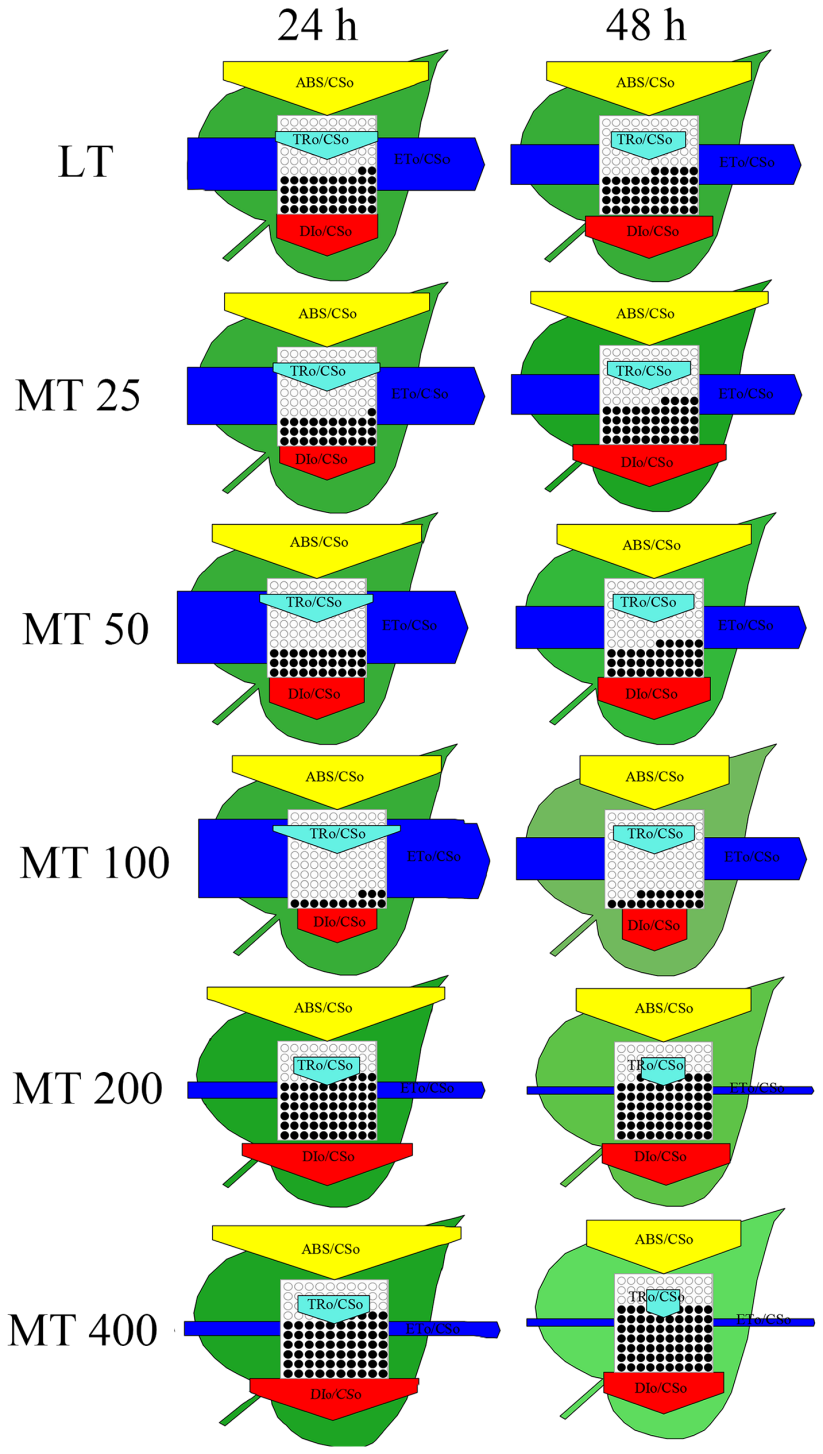
The energy flux models (leaf models) of low temperature-stressed cucumber plants pre-treated with different concentrations of melatonin (MT).

The energy pipeline models were developed to visualize and understand the symptoms of low temperature-stressed cucumber through analyzing the light absorption, trapping, electron transport, and dissipation of per excited cross section (CS_O_ = F_O_) (**Figure 7**). Results showed that MT 100 significantly improved the number of active RCs and light trapping. In addition, almost all energy fluxes were increased by MT 100 and decreased by MT 200 and MT 400. These results of the energy pipeline models were highly consistent with the values in **Figure 6**.

#### M_O_, Sm, and quantum yields or efficiencies/probabilities

**Figure 8:**
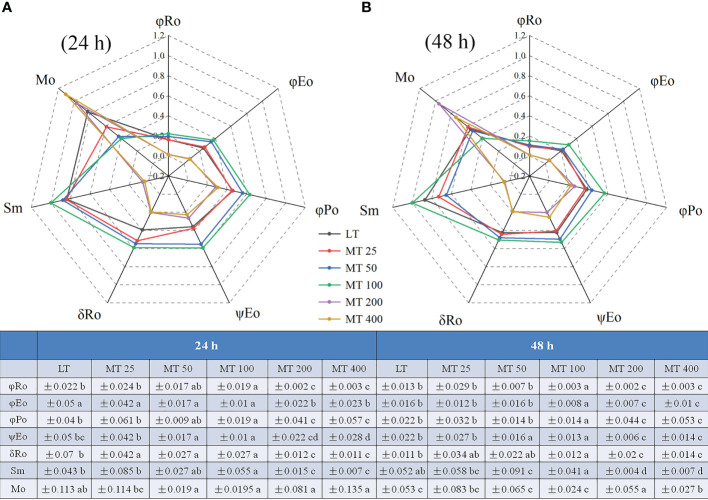
Radar plot of the JIP parameters of cucumber leaves under low temperature stress for 24 h **(A)** and 48 h **(B)**. The values were represented by the means ± SE. The same letters denoted that there is no significant difference at *P* < 0.05 according to Duncan’s test.

The relative value of the M_O_ and other chlorophyll fluorescence parameters are shown in **Figure 8**. Under low temperature conditions, different levels of MT had different effects on JIP parameter, and specific changes in different treatments were observed. For instance, the values of φ_Ro_, φ_Eo_, φ_Po_, ψ_Eo_, δ_Ro_, and Sm in MT 100-treated leaves were markedly higher than in LT-treated plants, while the Mo was obviously lower than in LT-treated plants. However, the MT 200 and MT 400 treatments showed the opposite effect to MT 100 when compared with LT (**Figures 8A, B** **)**.

### The modulated 820 nm reflection (MR_820_) signals and the parameters of low temperature-stressed cucumber plants pretreated with different levels of MT

**Figure 9:**
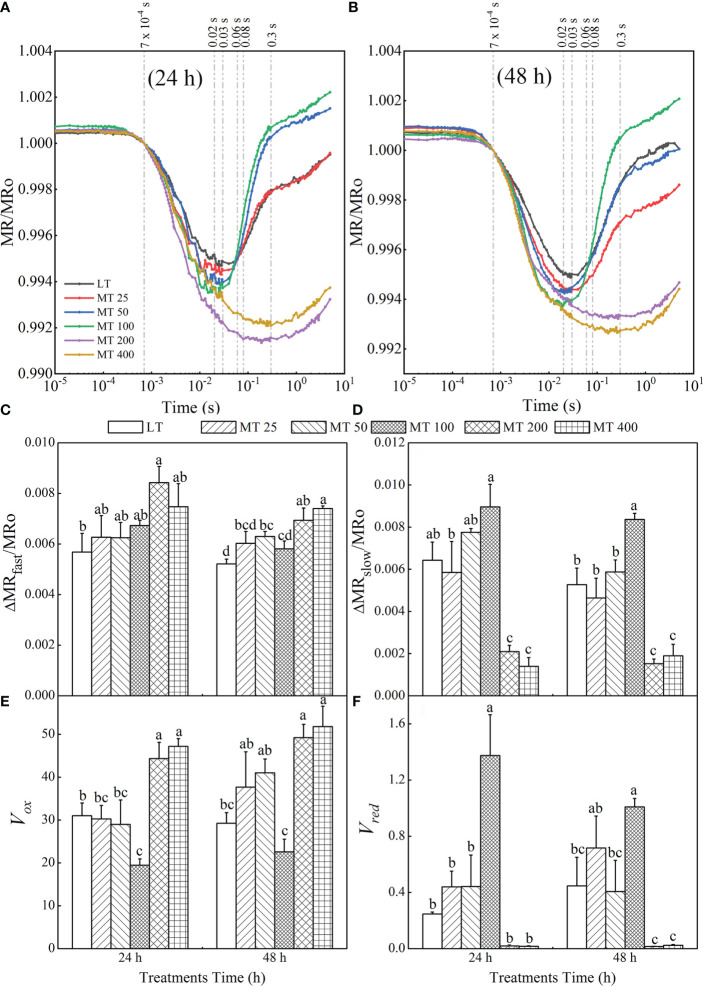
Effect of melatonin (MT) on the MR_820_ signal after low temperature stress for 24 h **(A)** and 48 h **(B)**. The time point 7×10^-4^ s represents the first reliable value of the MR/MRo (MR_O_) of each treatment; the time point 0.02 s represents the MR_min_ of MT 50 and MT 100 treatments, the time point 0.03s represents the MR_min_ of LT and MT 25 treatments, the time point 0.06s represents the end of *V_red_* in MT 50 and MT 100 treatments, the time point 0.08 s represents the end of *V_red_* in LT and MT 25 treatments, the time point 0.3 s represents the MR_min_ of MT 200 and MT 400 treatments, and the time point 0.6 s represents the end of *V_red_* in MT 200 and MT 400 treatments. The fast phase **(C)** was calculated as △MR_fast_/MR_O_ = |(MR_O_ – MR_min_)|/MR_O_. The slow phase **(D)** was calculated as △MR_slow_/MR_O_ =(MR_O_ – MR_max_)|/MR_O_. The oxidation rate of plastocyanin (PC) and PSI reaction center (P_700_) **(E)** was achieved: *V_ox_* = △MR/△t = (MR_2ms_ – MR_0.7 ms_)/(1.3 ms). The reduction rate of PC^+^ and P_700_^+^ **(F)** was calculated as *V_red_* = △MR/△t. The *V_red_* of LT and MT 25 treatments were calculated by *V_red_* = (MR_80ms_ – MR_30ms_)/(50 ms); The *V_red_* of MT 50 and MT 100 treatments were calculated by *V_red_* = (MR_60ms_ – MR_20ms_)/(40 ms); and The *V_red_* of MT 200 and MT 400 treatments were calculated by *V_red_* = (MR_600ms_ – MR_300ms_)/(300 ms). In this experiment, the MR of each treatment did not reach the maximum value, so the last value of MR was taken as MR_max_. The values were represented by the means ± SE. The same letters denoted that there is no significant difference at *P* < 0.05 according to Duncan’s test.

The MR_820_ signals normalized by MR_O_ (MR_0.7ms_) (MR/MR_O_) were used to further analyze the effect of MT on the PSI activity of low temperature-stressed cucumber seedlings (**Figures 9A, B** **)**. The rapid descent phase (oxidation of PC and P700) was induced by the two red-light pulses of M-PEA, indicating that the slow rise phase (re-reduction of PC^+^ and P_700_^+)^ would be later inducted in electrons transport from PSII. Under low temperature stress, different MT treatments led to the deformation of MR_820_ signals in cucumber seedlings, which showed changes in the lowest point of the rapid decline stage and in the highest point of the slow rise stage (**Figures 9A, B** **)**. Compared with LT, different MT treatments significantly decreased the lowest point of the oxidation phase of cucumber seedlings. In addition, the time reaching the lowest point of the oxidation phase was also advanced by the MT 50 and MT 100 treatments, while delayed by the MT 200 and MT 400 treatments when compared with LT treatment. The highest point of the re-reduction phase was also changed by different MT treatments. Compared with LT, MT 50 and MT 100 treatments significantly increased, while MT 200 and MT 400 treatments significantly decreased the highest point of the re-reduction phase after low temperature stress for 24 h, and MT 100 significantly increased, while the other treatments significantly decreased the highest point of re-reduction phase under low temperature stress for 48 h (**Figures 9A, B** **)**. These results indicated that the appropriate concentration of MT (MT 50 and MT 100) can enhance the redox capacity of PSI.

Based on the MR_820_ transient, several parameters derived from MR_820_ signals including △MR_fast_/MR_O_, △MR_slow_/MR_O_, PC and P700 oxidation rate (*V_ox_*) as well as the re-reduction rate of PC^+^ and P_700_^+^ (*V_red_*) were proposed in **Figures 9C–F**. The fast and slow phases can be quantified, respectively as △MR_fast_/MR_O_ and △MR_slow_/MR_O_. Compared with LT, different concentrations of MT treatments increased distinctly the values of △MR_fast_/MR_O_ at different levels (**Figure 9C**). On the other hand, MT 100 treatments led to a significant rise, while MT 200 and MT 400 treatments led to a significant decrease of △MR_slow_/MR_O_ and there was no obvious difference between LT and MT 25 or LT and MT 50 treatments (**Figure 9D**). *V_ox_* and *V_red_* were used to represent the oxidation of PC and P700 and reduction of PC^+^ and P_700_^+^, respectively. It is clear that MT 100 decreased *V_ox_* by 51.7% and 22.82% relative to LT after 24 h and 48 h of low temperature stress, respectively. There were no obvious changes in *V_ox_* after MT 25 and MT 50 treatment when compared with LT (**Figure 9E**). With MT 100 treatment, the value of *V_red_* was increased by 457.43% and 125.75% relative to LT for 24 h and 48 h, respectively. There was no obvious difference between LT, MT 25, and MT 50 treatment. Meanwhile, the value of *V_red_* in MT 200- and MT 400- treated leaves declined close to zero (**Figure 9F**).

## Discussion

Photosynthetic in plants starts from the light-harvesting systems. The part of the energy used for photochemical reaction drives the electron transport along with the thylakoid membrane of chloroplasts, and eventually produces ATP and NADPH as the energy of the Calvin-Benson cycle and photorespiratory cycle (Heber et al., 1978; Heber and Walker, 1992). The prompt fluorescence (OJIP) and modulated 820-nm reflection (MR_820_) can reflect all the changes in photochemical reactions because of the close connection with the photochemical reaction and heat dissipation (Zhu et al., 2005; Murchie and Lawson, 2013). Using the OJIP and MR_820_ signals, researchers have revealed the cultivar differences under chilling or heat stress, and the adverse effects of abiotic stresses including temperature, salinity, and drought, as well as the beneficial effect of exogenous signal molecules on photosynthesis, growth and development of plants (Kan et al., 2017; Zushi and Matsuzoe, 2017; Ahammed et al., 2018; Hu et al., 2018; Snider et al., 2018; Zhou et al., 2019; Chen et al., 2021). As a common environmental factor, low temperature stress seriously affects crop productivity by influencing plant growth and development (Ding et al., 2019). In this study, we applied MT in cucumber plants to study the changes in the photosynthetic electron transport chain and energy distribution by using OJIP and MR_820_ signals and attempted to explain how MT improved the adaptability of cucumber plants to low temperature stress.

As an antistress agent, MT has been reported against a number of abiotic stressors including low temperature (Arnao and Hernandez-Ruiz, 2015). Consistent with this, we found that MT 100 had a positive effect on plant phenotype, while the high concentration of MT (more than 200 μmol · L^-1^) aggravated the damage of low temperature stress to cucumber seedlings (**Figures 1A, B** **)**. A previous study showed that MT regulated low temperature tolerance of cucumbers by activating the antioxidant enzymes and inducing the key genes related to PSI, PSII and carbon assimilation (Zhang et al., 2021). The Mo represents the rate of closing PSII RCs (Guo et al., 2020). In our study, we also found that appropriate concentrations of MT could improve the activity of PSII of cucumber plants (Fv/Fm, Fv/Fo, PIabs) mainly by increasing the Mo under low temperature stress (**Figures 1**, **8**). The energy absorbed by plants drives electrons forward along the electron transport chain (Heber et al., 1978). The J-step (*V_J_*) increase indicates that the D1 protein is damaged and the electron transport from the primary quinone acceptor (Q_A_) to the secondary receptor quinone (Q_B_) is blocked, resulting in a large accumulation of Q_A_ **^-^** in RCs of PSII (Oukarroum et al., 2004; Guo et al., 2020). Our results demonstrated that the *V_J_* was significantly decreased by MT 50 and MT 100, suggesting that appropriate concentrations of MT (MT 50 and MT 100) could effectively protect D1 protein and promote electron transport.

We further analyzed OJIP and MR_820_ transients using the JIP-test method to investigate the mechanism of MT-induced changes in the electron transport chain of cucumber plants under low temperature stress. Generally, the OJIP transient shows polyphasic steps including O (Fo, at 20 μs with M-PEA, all RCs open), J (~2 ms), I (~30 ms) and P (Fm, maximal fluorescence yield) (Strasser et al., 1995; Strasser et al., 2004). However, other steps such as K- and L-step between O and J, G- and H-steps between I and P also appear in certain conditions (Strasser et al., 2004; Chen et al., 2016; Xia et al., 2019). Similarly, a study reported by Stirbet and Govindjee (2012) showed that the J- and I-step did not always appear at 2 ms and 30 ms, which might move to another position with different stress conditions. Compared with the traditional positions of J, I and P points, the positions of these three points lagged slightly (J point for 3 ms, I point for 80 ms, and P point did not reach the maximum value in our study) in our study (**Figure 2**). Furthermore, the structure and order of light-harvesting-complexes can be reflected by *F_O_* to a certain extent (Guo et al., 2020). Our study found that OJIP transient is sensitive to MT under low temperature stress. The OJIP transient was steep in MT 25- and MT 100-treated leaves than that in the LT, because of the increases from J-step to P-step at 24 h of low temperature stress (**Figure 2A**). The *F_O_* was increased by MT at 24 h of low temperature stress, while decreased by MT at 48 h of low temperature stress (**Figures 2A, B** **)**. The characteristics of the OJIP curve were most obvious in the MT 100 treatment, because the MT 100 treatment significantly reduced the O-step at 48 h of low temperature (**Figure 2B**). These findings indicated that MT mainly regulates the RCs of PSII under 24 h of low temperature stress, and with the extension of stress (48 h), MT can enhance the cucumber tolerance to low temperature by regulating energy capture efficiency of PSII, of which 100 μmol · L^-1^ MT (MT 100) had the best remission effect. The OJIP curve of MT 200- and MT 400-treated plants showed an increase after J-step, resulting in the disappearance of the IP phase (**Figure 2**). These results are highly consistent with **Figure 1F**. Combined with the previous research that reported the state of light absorption, chloroplast damage, and the activity response centers of PSII that can be partly reflected by the *F_O_*, *F_M_* and *V_J_* (Strasser et al., 2010), we concluded that MT 100 could regulate the energy absorption by regulating the internal structure of light-harvesting-complexes and protect PSII donor end deterioration caused by low temperature, thereby promoting the capacity of the PSII donor end to provide electrons due to an increase in the opened RCs of PSII.

From the L-band and K-band, we can understand the group of the PSII subunits or energetic connectivity between the antenna and RCs of PSII and the situation of OEC centers at the PSII donor side (Strasser et al., 2004; Kalaji et al., 2018). Studies showed that the K-band usually occurred in plants that suffer from chilling, heat or drought stress (Strasser et al., 2004; Chen et al., 2016; Silva Dalberto et al., 2017; Dimitrova et al., 2020; Zeng et al., 2022). This phenomenon might be indirectly caused by the block of PSII electron flow beyond Q_A_, resulting in a large accumulation of reactive oxygen species (ROS) in PSII (Rutherford and Krieger-Liszkay, 2001; Guo et al., 2020). In addition, the G-band represented the size of the PSI terminal electron acceptor pool. Furthermore, the maximal amplitude of the *W_O-I_* ≥ 1 curve is negatively correlated with the pool size of the terminal electron receptor on the PSI receptor side (Guo et al., 2020). Here, MT 100 induced a decrease in L-band, K-band, as well as G-band and an increase in OEC centers, Sm, and the maximal amplitude of the *W_O-I_* ≥ 1 curve (the IP phase), (**Figures 3**, **4**, **5A, B**, and **8**). These results corroborated that MT 100 increased the low temperature tolerance of cucumber by enhancing the connectivity between PSII antenna pigment and PSII reaction center, protecting the fraction of the OEC activity, increasing the electron transfer rate, and repairing the electron acceptor pool at the receptor side of PSI terminal, thereby promoting PSII electron flow beyond Q_A_.

JIP-test has been demonstrated to reveal the stepwise flow of energy through PSII (Strasser et al., 2004; Guo and Tan, 2015; Tsimilli-Michael, 2020). According to the energy absorption, capture and transfer, it is clear that MT changed the multiple sites of the electron transport chain of low temperature-stressed cucumber plants. Previous research has shown that iron deficiency and saline-alkali stress induced the increase of ABS/RC, which indicates that part of PSII RCs is inactivated (Kalaji et al., 2014). Our study showed the ABS/RC, TRo/RC, and DIo/RC were significantly lower in MT 100-treated plants than in LT treatment. However, the light energy was used mainly for transfer (ET_O_/RC, RE_O_/RC) and beyond, and less for capture (TR_O_/RC) and dissipation (DI_O_/RC), which explains the high efficiency parameters related to quantum yields (φ_Po_, φ_Eo_, φ_Ro_) (**Figure 8**). This is consistent with the conclusion presented by Shomali et al. (2021), who suggested that MT protected the photosynthetic apparatus and further improved the photosynthetic performance (Shomali et al., 2021). In other words, MT 100 can enhance the low temperature tolerance of cucumber seedlings by activating part of PSII reaction centers, reducing energy absorption and capture, enhancing energy transfer in the PSII and improving light energy utilization. Coincidentally, the leaf energy flux models (**Figure 7**) also confirm these results. Electron transport (ET) is more sensitive to low temperature than excitation energy capture (TR). MT 100 induced the higher values of ETo/TR and ψ_Eo_ (**Figures 6**, **8**) possibly because energy was activated at ET by MT under low temperature conditions, which might be the main reason for the increase of φ_Ro_. Furthermore, δ_Ro_ was different between LT and MT treatments (**Figure 8**), which meant that RE was affected by MT under low temperature stress. MT 100 significantly reduced ABS/RC and DIo/RC, while increased ETo/RC and REo/RC (**Figures 6**, **7**). This may be because the photosystem electron transfer chain of cucumber leaves is partly recovered by MT 100 under low temperature conditions. These suggested that MT protected the photosynthetic machinery, increased the utilization of captured energy for the photochemical reaction, greatly reduced the excitation pressure on the RC and allowed smoother energy flow.

Our results also revealed that MT had a vital impact on PSI. The MR_820_ signal can reflect the electron transport and the redox state of PC and P700 in PSI (Gao et al., 2014; Hamdani et al., 2015; Guo et al., 2020). Accumulation of PC^+^ and P_700_^+^ results in a fast decrease in MR/MR_O_ (fast phase), which can be expressed as △MR_fast_/MR_O_. The minimal MR/MR_O_ is a relatively stable state, where the oxidation rate is equal to the reduction rates of PC and P700. Subsequently, electrons coming from P_680_ arrive at P_700_^+^ and PC^+^, where they are oxidized, that is, P_700_^+^ and PC^+^ are re-reduced, causing an increased stage in MR/MR_O_ (slow phase), which can be represented by △MR_slow_/MR_O_ (Strasser et al., 2010). The minimal of MR/MR_O_ was decreased by MT (**Figures 9A, B** **)**, whereas the △MR_fast_/MR_O_ was gradually increased by MT at low temperature conditions (**Figure 9C**). In addition, the time reaching to the lowest point of the oxidation phase was obviously advanced by the MT 50 and MT 100 treatments, while delayed by the MT 200 and MT 400 treatments when compared with LT treatment. These indicated the faster oxidation rates of P700 and PC, and the photochemical activity of PSI was enhanced by MT under low temperature stress. Obviously, the MT had an essential effect on the slow phase of the MR_820_ signals (**Figure 9D**). The slow rising phase of MT 100-treated samples significantly increased, while almost disappeared in MT 200- and MT 400-treated plants in the MR_820_ signal (**Figures 9A, B** **)**. Our results were highly consistent with Zhang et al. (2021). These results suggested that the MT 100 could improve entirely PSII electron flow *via* Q_A_ to PC^+^ and P_700_^+^. The *V*_ox_ and *V*_red_ were used to further quantify the redox rate of PC and P700. The traditional *V*_ox_ and *V*_red_ were calculated in two particular time ranges, 0.7–3 ms (fast phase) and 7–300 ms (slow phase), respectively (Gao et al., 2014). However, the MR/MR_O_ signal vs. linear time scale of these two particular time ranges is not a straight line. So, the new time ranges from 0.7 to 2 ms (*V*_ox_) were proposed for the calculation of *V*_ox_ in our study (**Figure 9E**). In addition, for the *V*_red_, the appearance of the lowest point of MR/MR_O_ kinetics is different for each treatment under low temperature stress. So analysis at the new particular time was carried out and the calculation formulas were presented in **Figure 9**. In this study, the *V*_ox_ was limited by MT 100, while *V*_red_ was improved by MT 100 under low temperature stress. This may be because MT 100 connects or increases the core complexes and electron transporters of PSI, thereby allowing more electrons to flow to PSI to reduce P_700_^+^ and PC^+^ under low temperature stress (Zhou et al., 2019). The reduced oxidation rate of PC and P700 and the increased reduction rate of PC^+^ and P_700_^+^ by MT 100 make the electron transfer in the photosynthetic mechanism smoother, and then improve the photosynthesis of cucumber seedlings at low temperature conditions. The reduction activity of PSI can result from the capacity of pumping electrons to the intersystem electron transport chain by PSII (Kan et al., 2017), the connection state between PSII and PSI, and the improvement of the PSI acceptor side (Dąbrowski et al., 2021). Based on these studies and our analysis of the OJIP, MR_820_ signal, and related JIP-test parameters, we conclude that MT could regulate the multiple sites of the photosynthetic electron transport chain and increase the PSII activity and electron transfer capacity under low temperature stress.

## Conclusions

**Figure 10:**
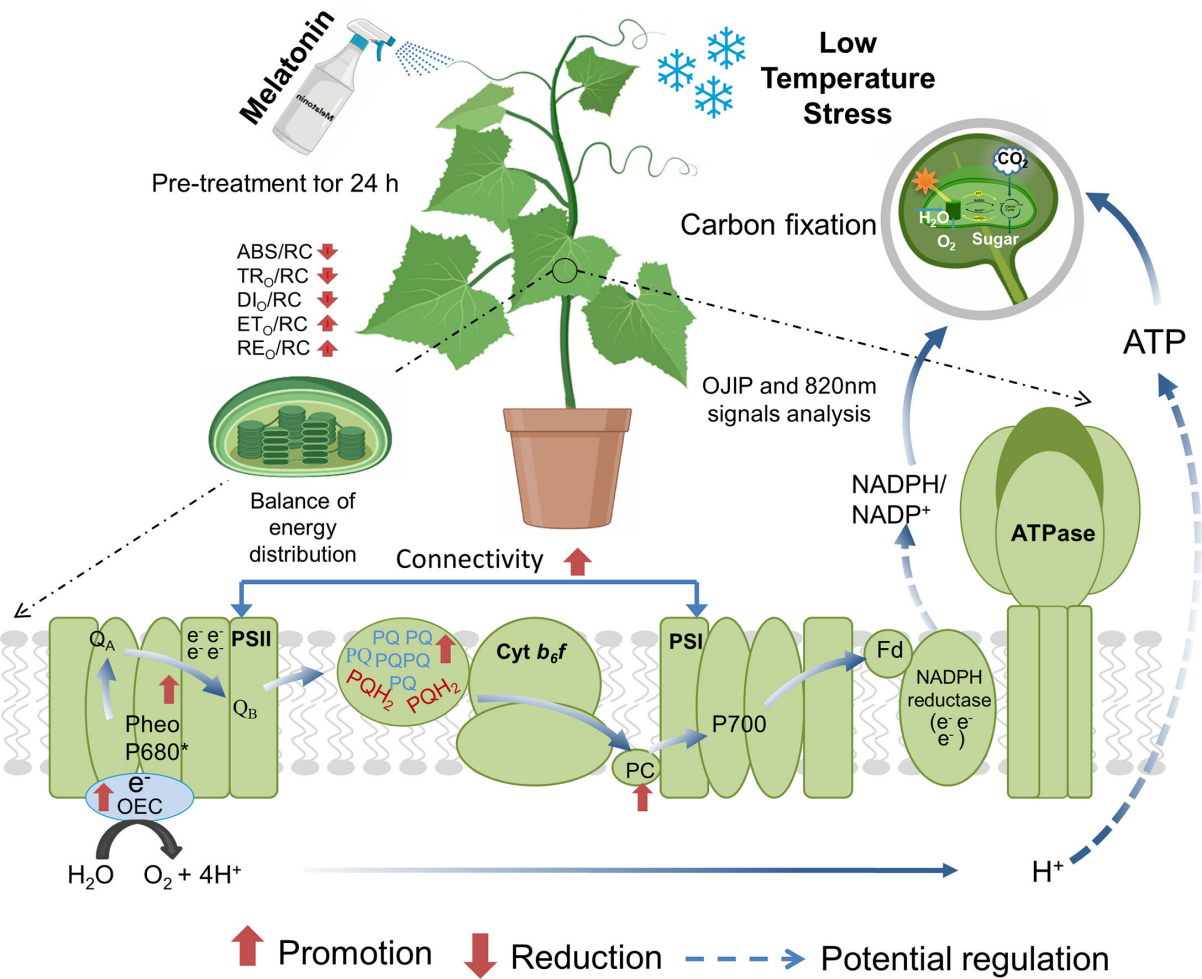
A hypothetical model showing melatonin (MT)-induced regulation of photosynthesis and photosystem performance under low temperature stress in cucumber. MT could improve light reactions and electron transport from PSII *via* Q_A_ to PC^+^ and P700^+^ in the photosystem by strengthening the connectivity between PSI and PSII. MT also improved the OCE activity, resulting in an increase in the photochemical decomposition of water and more H^+^ drives ATP synthesis *via* ATP synthase. In addition, MT increased the PSI activity, deoxidation rates of PC^+^ and P700^+^ and decreased the oxidation rate of PC and P700 and further increased the electron transport.  QA, primary quinone electron acceptor; PSII, photosystem II; PSI, photosystem I; OCE, oxygen evolving complex; PC, plastocyanin; P700, PSI reaction center.

Low temperature stress damaged the effectiveness of photosynthesis, which was manifested by severely inhibited photosystem performance and impaired plant phenotype. Foliar application of MT before low temperature stress can induce the efficiency of PSII (Fv/Fm and Fv/Fo), the performance of the photosystem II donor/acceptor side (PIabs, *W_K_* and *V_J_*), the activity of PSI (*W_OI_* ≥ 1), redox rate of PSI (*V*_ox_ and *V*_red_), the balance of the energy distribution (ABS/RC, TR_O_/RC, DI_O_/RC, ET_O_/RC and RE_O_/RC), and the quantum yields (*φ*_Po_, *φ*_Eo_, *φ*_Ro_, ψ_Eo_ and *δ*_Ro_) of cucumber leaves, thus repairing the photosynthetic electron transport chain under low temperature stress. We conclude that an appropriate concentration of MT (100 μmol · L^-1^) is beneficial for the improvement of the connectivity between PSI and PSII and the performance of electron transfer and energy distribution in cucumber leaves, which result from the MT-induced regulation of multiple sites of the photosynthetic electron transport chain, and potential synthesis of more energy (ATP and NADPH) under low temperature stress (**Figure 10**). However, high concentrations of MT (≥ 200 μmol · L^-1^) showed completely negative effects on low temperature tolerance in cucumber plants.

## Data availability statement

The original contributions presented in the study are included in the article/**Supplementary Material**. Further inquiries can be directed to the corresponding authors.

## Author contributions

This work was carried out in collaboration between all the authors. PW, JXC, and HL conceived designed the experiments. PW, YM, BH, JYC, WW, and YZ performed the experiments, analyzed the data, prepared figures and/or tables. PW and YM wrote the original draft. JXC, GA, HL, HC, and WX reviewed and edited the manuscript. All authors reviewed drafts of the paper, and approved the final manuscript.

## Funding

We are grateful to the National Natural Science Foundation of China, China (No. 31560571) for providing financial support to our research.

## Conflict of interest

The authors declare that the research was conducted in the absence of any commercial or financial relationships that could be construed as a potential conflict of interest.

## Publisher’s note

All claims expressed in this article are solely those of the authors and do not necessarily represent those of their affiliated organizations, or those of the publisher, the editors and the reviewers. Any product that may be evaluated in this article, or claim that may be made by its manufacturer, is not guaranteed or endorsed by the publisher.

## References

[B1] Ahammed G. J. Xu W. Liu A. Chen S. (2018). *COMT1* silencing aggravates heat stress-induced reduction in photosynthesis by decreasing chlorophyll content, photosystem II activity, and electron transport efficiency in tomato. Front. Plant Sci. 9. doi: 10.3389/fpls.2018.00998 PMC6056654 30065736

[B2] Arnao M. B. Hernández-Ruiz J. (2014). Melatonin: plant growth regulator and/or biostimulator during stress? Trends Plant Sci. 19, 789–797. doi: 10.1016/j.tplants.2014.07.006 25156541

[B3] Arnao M. B. Hernandez-Ruiz J. (2015). Functions of melatonin in plants: a review. J. Pineal Res. 59, 133–150. doi: 10.1111/jpi.12253 26094813

[B4] Chen S. Yang J. Zhang M. Strasser R. J. Qiang S. (2016). Classification and characteristics of heat tolerance in *Ageratina adenophora* populations using fast chlorophyll a fluorescence rise O-J-I-P. Environ. Exp. Bot. 122, 126–140. doi: 10.1016/j.envexpbot.2015.09.011

[B5] Chen X. Zhou Y. Cong Y. Zhu P. Xing J. Cui J.. (2021). Ascorbic acid-induced photosynthetic adaptability of processing tomatoes to salt stress probed by fast OJIP fluorescence rise. Front. Plant Sci. 12. doi: 10.3389/fpls.2021.594400 PMC8415309 34484251

[B6] Chinnusamy V. Zhu J. K. Sunkar R. (2010). Gene regulation during cold stress acclimation in plants. Methods Mol. Biol. 639, 39–55. doi: 10.1007/978-1-60761-702-0_3 20387039 PMC3064467

[B7] Corpas F. J. (2019). Hydrogen sulfide: a new warrior against abiotic stress. Trends Plant Sci. 24, 983–988. doi: 10.1016/j.tplants.2019.08.003 31494025

[B8] Cui J. Zhou Y. Ding J. Xia X. Shi K. Chen S.. (2011). Role of nitric oxide in hydrogen peroxide-dependent induction of abiotic stress tolerance by brassinosteroids in cucumber. Plant Cell Environ. 34, 347–358. doi: 10.1111/j.1365-3040.2010.02248.x 21054437

[B9] Dąbrowski P. Baczewska-Dąbrowska A. H. Bussotti F. Pollastrini M. Piekut K. Kowalik W.. (2021). Photosynthetic efficiency of microcystis ssp. under salt stress. Environ. Exp. Bot. 186, 104459. doi: 10.1016/j.envexpbot.2021.104459

[B10] Debnath B. Islam W. Li M. Sun Y. Lu X. Mitra S.. (2019). Melatonin mediates enhancement of stress tolerance in plants. Int. J. Mol. Sci. 20, 1040. doi: 10.3390/ijms20051040 PMC6429401 30818835

[B11] Dimitrova S. Paunov M. Pavlova B. Dankov K. Kouzmanova M. Velikova V.. (2020). Photosynthetic efficiency of two *Platanus orientalis* l. ecotypes exposed to moderately high temperature –JIP–test analysis. Photosynthetica 58, 657–670. doi: 10.32615/ps.2020.012

[B12] Ding Y. Shi Y. Yang S. (2019). Advances and challenges in uncovering cold tolerance regulatory mechanisms in plants. New Phytol. 222, 1690–1704. doi: 10.1111/nph.15696 30664232

[B13] Ensminger I. Busch F. Huner N. P. A. (2006). Photostasis and cold acclimation: sensing low temperature through photosynthesis. Physiol. Plant 126, 28–44. doi: 10.1111/j.1399-3054.2006.00627.x

[B14] Fan J. Hu Z. Xie Y. Chan Z. Chen K. Amombo E.. (2015). Alleviation of cold damage to photosystem II and metabolisms by melatonin in bermudagrass. Front. Plant Sci. 6. doi: 10.3389/fpls.2015.00925 PMC4630300 26579171

[B15] Feng Y. Fu X. Han L. Xu C. Liu C. Bi H.. (2021). Nitric oxide functions as a downstream signal for melatonin-induced cold tolerance in cucumber seedlings. Front. Plant Sci. 12. doi: 10.3389/fpls.2021.686545 PMC8343141 34367212

[B16] Gao J. Li P. Ma F. Goltsev V. (2014). Photosynthetic performance during leaf expansion in *Malus micromalus* probed by chlorophyll a fluorescence and modulated 820nm reflection. J. Photochem. Photobiol. B-Biol. 137, 144–150. doi: 10.1016/j.jphotobiol.2013.12.005 24373888

[B17] Guo Y. Lu Y. Goltsev V. Strasser R. J. Kalaji H. M. Wang H.. (2020). Comparative effect of tenuazonic acid, diuron, bentazone, dibromothymoquinone and methyl viologen on the kinetics of chl *a* fluorescence rise OJIP and the MR820 signal. Plant Physiol. Biochem. 156, 39–48. doi: 10.1016/j.plaphy.2020.08.044 32906020

[B18] Guo Y. Tan J. (2015). Recent advances in the application of chlorophyll *a* fluorescence from photosystem II. Photochem. Photobiol. 91, 1–14. doi: 10.1111/php.12362 25314903

[B19] Hamdani S. Qu M. Xin C. P. Li M. Chu C. Govindjee. (2015). Variations between the photosynthetic properties of elite and landrace Chinese rice cultivars revealed by simultaneous measurements of 820 nm transmission signal and chlorophyll *a* fluorescence induction. J. Plant Physiol. 177, 128–138. doi: 10.1016/j.jplph.2014.12.019 25732386

[B20] Heber U. Egneus H. Hanck U. Jensen M. K6ster S. (1978). Regulation of photosynthetic electron transport and photophosphorylation in intact chloroplasts and leaves of *Spinacia oleracea* l. Planta 143, 41–19. doi: 10.1007/BF00389050 24408259

[B21] Heber U. Walker D. (1992). Concerning a dual function of coupled cyclic electron transport in leaves. Plant Physiol. 100, 1621–1626. doi: 10.1104/pp.100.4.1621 16653176 PMC1075843

[B22] Hu W. Snider J. L. Chastain D. R. Slaton W. Tishchenko V. (2018). Sub-Optimal emergence temperature alters thermotolerance of thylakoid component processes in cotton seedlings. Environ. Exp. Bot. 155, 360–367. doi: 10.1016/j.envexpbot.2018.07.020

[B23] Jahan M. S. Guo S. Sun J. Shu S. Wang Y. El-Yazied A. A.. (2021). Melatonin-mediated photosynthetic performance of tomato seedlings under high-temperature stress. Plant Physiol. Biochem. 167, 309–320. doi: 10.1016/j.plaphy.2021.08.002 34392044

[B24] Kalaji H. M. Bąba W. Gediga K. Goltsev V. Samborska I. A. Cetner M. D.. (2018). Chlorophyll fluorescence as a tool for nutrient status identification in rapeseed plants. Photosynth. Res. 136, 329–343. doi: 10.1007/s11120-017-0467-7 29185137 PMC5937862

[B25] Kalaji H. M. Oukarroum A. Alexandrov V. Kouzmanova M. Brestic M. Zivcak M.. (2014). Identification of nutrient deficiency in maize and tomato plants by in vivo chlorophyll a fluorescence measurements. Plant Physiol. Biochem. 81, 16–25. doi: 10.1016/j.plaphy.2014.03.029 24811616

[B26] Kan X. Ren J. Chen T. Cui M. Li C. Zhou R.. (2017). Effects of salinity on photosynthesis in maize probed by prompt fluorescence, delayed fluorescence and P700 signals. Environ. Exp. Bot. 140, 56–64. doi: 10.1016/j.envexpbot.2017.05.019

[B27] Khan T. A. Fariduddin Q. Nazir F. Saleem M. (2020). Melatonin in business with abiotic stresses in plants. Physiol. Mol. Biol. Plants 26, 1931–1944. doi: 10.1007/s12298-020-00878-z 33088040 PMC7548266

[B28] Krieger-Liszkay A. Shimakawa G. (2022). Regulation of the generation of reactive oxygen species during photosynthetic electron transport. Biochem. Soc Trans. 50, 1025–1034. doi: 10.1042/BST20211246 35437580

[B29] Lee H. J. Lee J. H. Wi S. Jang Y. An S. Choi C. K.. (2021). Exogenously applied glutamic acid confers improved yield through increased photosynthesis efficiency and antioxidant defense system under chilling stress condition in *Solanum lycopersicum* l. cv. dotaerang dia. Sci. Hortic. 277, 109817. doi: 10.1016/j.scienta.2020.109817

[B30] Li H. Guo Y. Lan Z. Xu K. Chang J. Ahammed G. J.. (2021). Methyl jasmonate mediates melatonin-induced cold tolerance of grafted watermelon plants. Hortic. Res. 8, 57. doi: 10.1038/s41438-021-00496-0 33750773 PMC7943586

[B31] Liu X. Zhou Y. Xiao J. Bao F. (2018). Effects of chilling on the structure, function and development of chloroplasts. Front. Plant Sci. 9. doi: 10.3389/fpls.2018.01715 PMC6262076 30524465

[B32] Murchie E. H. Lawson T. (2013). Chlorophyll fluorescence analysis: a guide to good practice and understanding some new applications. J. Exp. Bot. 64, 3983–3998. doi: 10.1093/jxb/ert208 23913954

[B33] Oukarroum A. Strasser R. J. Staden J. V. (2004). Phenotyping of dark and light adapted barley plants by the fast chlorophyll *a* fluorescence rise OJIP. S. Afr. J. Bot. 70, 277–283. doi: 10.1016/S0254-6299(15)30246-5

[B34] Ploschuk E. L. Bado L. A. Salinas M. Wassner D. F. Windauer L. B. Insausti P. (2014). Photosynthesis and fluorescence responses of jatropha curcas to chilling and freezing stress during early vegetative stages. Environ. Exp. Bot. 102, 18–26. doi: 10.1016/j.envexpbot.2014.02.005

[B35] Reiter R. Tan D. X. Zhou Z. Cruz M. Fuentes-Broto L. Galano A. (2015). Phytomelatonin: assisting plants to survive and thrive. Molecules 20, 7396–7437. doi: 10.3390/molecules20047396 25911967 PMC6272735

[B36] Ruelland E. Vaultier M. N. Zachowski A. Hurry V. (2009). Cold signalling and cold acclimation in plants. Adv. Bot. Res. 49, 35–150. doi: 10.1016/s0065-2296(08)00602-2

[B37] Rutherford A. W. Krieger-Liszkay A. (2001). Herbicide-induced oxidative stress in photosystem II. Trends Biochem. Sci. 26, 648–653. doi: 10.1016/s0968-0004(01)01953-3 11701322

[B38] Schansker G. Srivastava A. Govindjee Strasser R. J. (2003). Characterization of the 820-nm transmission signal paralleling the chlorophyll a fluorescence rise (OJIP) in pea leaves. Funct. Plant Biol. 30, 785–796. doi: 10.1071/fp03032 32689062

[B39] Shikanai T. (2011). Regulation of photosynthetic electron transport. Biochim. Biophys. Acta 1807, 375–383. doi: 10.1016/j.bbabio.2010.11.010 21439931

[B40] Shomali A. Aliniaeifard S. Didaran F. Lotfi M. Mohammadian M. Seif M.. (2021). Synergistic effects of melatonin and gamma-aminobutyric acid on protection of photosynthesis system in response to multiple abiotic stressors. Cells 10, 1631. doi: 10.3390/cells10071631 34209882 PMC8306587

[B41] Silva Dalberto D. Garbin Martinazzo E. Antonio Bacarin M. (2017). Chlorophyll *a* fluorescence reveals adaptation strategies in drought stress in *Ricinus communis* . Braz. J. Bot. 40, 861–870. doi: 10.1007/s40415-017-0412-1

[B42] Snider J. L. Thangthong N. Pilon C. Virk G. Tishchenko V. (2018). OJIP-fluorescence parameters as rapid indicators of cotton (*Gossypium hirsutum* l.) seedling vigor under contrasting growth temperature regimes. Plant Physiol. Biochem. 132, 249–257. doi: 10.1016/j.plaphy.2018.09.015 30237089

[B43] Stirbet A. Govindjee (2011). On the relation between the kautsky effect (chlorophyll a fluorescence induction) and photosystem II: Basics and applications of the OJIP fluorescence transient. J. Photochem. Photobiol. B-Biol. 104, 236–257. doi: 10.1016/j.jphotobiol.2010.12.010 21295993

[B44] Stirbet A. Govindjee (2012). Chlorophyll a fluorescence induction: a personal perspective of the thermal phase, the J-I-P rise. Photosynth. Res. 113, 15–61. doi: 10.1007/s11120-012-9754-5 22810945

[B45] Strasser R. J. Srivastava A. Govindjee (1995). Polyphasic chlorophyll a fluorescence transient in plants and cyanobacteria. Photochem. Photobiol. 61, 32–42. doi: 10.1111/j.1751-1097.1995.tb09240.x

[B46] Strasser R. J. Tsimilli-Michael M. Qiang S. Goltsev V. (2010). Simultaneous *in vivo* recording of prompt and delayed fluorescence and 820-nm reflection changes during drying and after rehydration of the resurrection plant *Haberlea rhodopensis* . Biochim. Biophys. Acta 1797, 1313–1326. doi: 10.1016/j.bbabio.2010.03.008 20226756

[B47] Strasser R. J. Tsimilli-Michael M. Srivastava A. (2004). Analysis of the chlorophyll a fluorescence transient (Dordrecht: Springer), 321–362. doi: 10.1007/978-1-4020-3218-9_12

[B48] Sun C. Liu L. Wang L. Li B. Jin C. Lin X. (2020). Melatonin: A master regulator of plant development and stress responses. J. Integr. Plant Biol. 63, 126–145. doi: 10.1111/jipb.12993 32678945

[B49] Theocharis A. Clément C. Barka E. A. (2012). Physiological and molecular changes in plants grown at low temperatures. Planta 235, 1091–1105. doi: 10.1007/s00425-012-1641-y 22526498

[B50] Tiwari R. K. Lal M. K. Naga K. C. Kumar R. Chourasia K. N. Shivaramu. (2020). Emerging roles of melatonin in mitigating abiotic and biotic stresses of horticultural crops. Sci. Hortic. 272, 109592. doi: 10.1016/j.scienta.2020.109592

[B51] Tsimilli-Michael M. (2020). Revisiting JIP-test: An educative review on concepts, assumptions, approximations, definitions and terminology. Photosynthetica 58, 275–292. doi: 10.32615/ps.2019.150

[B52] Wang K. Xing Q. Ahammed G. J. Zhou J. (2022). Functions and prospects of melatonin in plant growth, yield and quality. J. Exp. Bot. 2022, erac233. doi: 10.1093/jxb/erac233 35640564

[B53] Wang F. Yan J. Ahammed G. J. Wang X. Bu X. Xiang H.. (2020). PGR5/PGRL1 and NDH mediate far-red light-induced photoprotection in response to chilling stress in tomato. Front. Plant Sci. 11. doi: 10.3389/fpls.2020.00669 PMC7270563 32547581

[B54] Wu P. Xiao C. Cui J. Hao B. Zhang W. Yang Z.. (2020). Nitric oxide and its interaction with hydrogen peroxide enhance plant tolerance to low temperatures by improving the efficiency of the calvin cycle and the ascorbate-glutathione cycle in cucumber seedlings. J. Plant Growth Regul. 40, 2390–2408. doi: 10.1007/s00344-020-10242-w

[B55] Xia Q. Tan J. Cheng S. Jiang Y. Guo Y. (2019). Sensing plant physiology and environmental stress by automatically tracking *F_j_ * and *F_i_ * features in PSII chlorophyll fluorescence induction. Photochem. Photobiol. 95, 1495–1503. doi: 10.1111/php.13141 31309566

[B56] Xiong D. Liu X. Liu L. Douthe C. Li Y. Peng S.. (2015). Rapid responses of mesophyll conductance to changes of CO_2_ concentration, temperature and irradiance are affected by n supplements in rice. Plant Cell Environ. 38, 2541–2550. doi: 10.1111/pce.12558 25923314

[B57] Zeng J. J. Hu W. H. Hu X. H. Tao H. M. Zhong L. Liu L. L. (2022). Upregulation of the mitochondrial alternative oxidase pathway improves PSII function and photosynthetic electron transport in tomato seedlings under chilling stress. Photosynthetica 60, 271–279. doi: 10.32615/ps.2022.019 PMC11558502 39650763

[B58] Zhang X. Feng Y. Jing T. Liu X. Ai X. Bi H. (2021). Melatonin promotes the chilling tolerance of cucumber seedlings by regulating antioxidant system and relieving photoinhibition. Front. Plant Sci. 12. doi: 10.3389/fpls.2021.789617 PMC8695794 34956288

[B59] Zhang Z. Wu P. Zhang W. Yang Z. Liu H. Ahammed G. J.. (2020). Calcium is involved in exogenous NO-induced enhancement of photosynthesis in cucumber (*Cucumis sativus* l.) seedlings under low temperature. Sci. Hortic. 261, 108953. doi: 10.1016/j.scienta.2019.108953

[B60] Zhao C. Yang M. Wu X. Wang Y. Zhang R. (2021). Physiological and transcriptomic analyses of the effects of exogenous melatonin on drought tolerance in maize (*Zea mays* l.). Plant Physiol. Biochem. 168, 128–142. doi: 10.1016/j.plaphy.2021.09.044 34628174

[B61] Zhou Y. Diao M. Cui J. X. Chen X. J. Wen Z. L. Zhang J. W.. (2018). Exogenous GSH protects tomatoes against salt stress by modulating photosystem II efficiency, absorbed light allocation and H_2_O_2_-scavenging system in chloroplasts. J. Integr. Agric. 17, 2257–2272. doi: 10.1016/S2095-3119(18)62068-4

[B62] Zhou R. Kan X. Chen J. Hua H. Li Y. Ren J.. (2019). Drought-induced changes in photosynthetic electron transport in maize probed by prompt fluorescence, delayed fluorescence, P700 and cyclic electron flow signals. Environ. Exp. Bot. 158, 51–62. doi: 10.1016/j.envexpbot.2018.11.005

[B63] Zhu X. G. Govindjee Baker N. R. deSturler E. Ort D. R. Long S. P. (2005). Chlorophyll *a* fluorescence induction kinetics in leaves predicted from a model describing each discrete step of excitation energy and electron transfer associated with photosystem II. Planta 223, 114–133. doi: 10.1007/s00425-005-0064-4 16411287

[B64] Zushi K. Matsuzoe N. (2017). Using of chlorophyll a fluorescence OJIP transients for sensing salt stress in the leaves and fruits of tomato. Sci. Hortic. 219, 216–221. doi: 10.1016/j.scienta.2017.03.016

